# The Utility of Tranexamic Acid in Endoscopic Surgeries for Benign Prostatic Hyperplasia

**DOI:** 10.1007/s11934-025-01271-7

**Published:** 2025-05-17

**Authors:** Nicolas Siron, David C. Dalton, Marcelino Rivera, T. Max Shelton

**Affiliations:** Department of Urology, Indiana University School of Medicine, Indianapolis, IN USA

**Keywords:** Tranexamic acid, BPH, Endoscopic surgery

## Abstract

**Purpose of Review:**

Tranexamic acid (TXA) is an anti-fibrinolytic agent that prevents degradation of fibrin by blocking the ability of plasminogen to bind to fibrin and the proteolytic activity of plasmin. TXA has been proven to be useful in reducing bleeding complications in multiple types of surgery. In this article, we will review the current usage of TXA in endoscopic surgeries for benign prostatic hyperplasia (BPH).

**Recent Findings:**

The use of TXA for endoscopic BPH surgeries has mainly been studied for transurethral resection of the prostate (TURP). In the clinical trials assessing the use of TXA and TURP, TXA demonstrated reduced intraoperative bleeding independent of administration route. However, this did not consistently translate to reduced hospitalization or catheterization times. Evidence for the use of TXA and holmium laser enucleation of the prostate (HoLEP) has begun to emerge, and to date limited benefit has been demonstrated. This result is likely due to the excellent innate hemostatic control associated with the procedure. However, further studies are required to validate these findings. With recent innovation in new types of endoscopic BPH surgeries, the benefit of TXA during other types of BPH procedures also require more study.

**Summary:**

Within the context of endoscopic surgeries for BPH, TXA appears to have the most benefit when performing TURP. More evidence is required to conclude on the benefit in other types of BPH surgery including HoLEP.

## Introduction

Tranexamic acid (TXA) is a synthetic lysine amino acid derivative that reversibly binds to plasminogen preventing it from binding to fibrin as well as exerting an inhibitory effect on plasmin proteolytic activity [1–3]. Through these two mechanisms TXA prolongs the thrombin time thus acting as an anti-fibrinolytic agent. Current formulations of TXA allow it to be administered intravenously (IV) or orally. TXA is mainly eliminated through urinary excretion via glomerular filtration. The half-life of TXA administered intravenously is about 2 h. TXA has a favourable safety profile with no known carcinogenic or genotoxic effects. In terms of adverse effects, TXA may increase the risk of thromboembolic events and is associated with seizures, dizziness, and visual disturbances. However, the latter adverse reaction is only based on animal studies. Given its relatively safety profile, there are few contraindications for TXA. These contraindications include patients with hypersensitivity to TXA, visual disturbances, history of venous or arterial thromboembolism, active thromboembolic disease, and intracranial bleeding [1–3]. 

## TXA and Surgical Procedures

Currently, the Food and Drug Administration (FDA) has approved the usage of TXA for bleeding prevention in hemophiliacs in the context of tooth extraction and menorrhagia [1]. Despite this, substantial evidence supports the off-label use of TXA in the context of trauma and different types of surgery. The use of TXA in the context of trauma was largely supported by the seminal CRASH-2 trial published in 2013. The CRASH-2 trial was a randomised placebo-controlled trial that studied the use of IV TXA in the prevention of bleeding in trauma patients actively bleeding or at risk of significant bleeding [4]. A total of 20,211 adult trauma patients were recruited and equally randomized. The CRASH-2 trial demonstrated that IV TXA significantly reduced the risk of death in bleeding trauma patients if administered within approximately 3 h. However, when considering the benefit of TXA in isolated blunt organ injury such as renal trauma, the improvement in outcomes is less unequivocal. Kovalev et al. published a retrospective study in 2023 demonstrating little difference in mortality and need for radiological intervention when TXA is administered in the context of isolated blunt organ injury [5]. 

The impetus of the CRASH-2 and similar trials largely stemmed from building evidence proving that TXA and other antifibrinolytic drugs administered during surgery reduced perioperative blood loss and the need for red blood cell transfusions [6, 7]. In 2012, Ker et al. published a meta-analysis on the effect of TXA on surgical bleeding [6]. In this review, they analyzed 10 488 patients across 129 randomized control trials (RCT) covering cardiac, orthopedic, gynecological and other types of surgery. In their analysis, TXA reduced blood transfusions (RR 0.62, *p* < 0.001) and deaths (RR 0.61, *p* = 0.04) by more than a third. The effect of TXA on risk for myocardial infarction (MI), stroke, deep venous thrombosis (DVT), and pulmonary embolism (PE) was equivocal. More recent data also supports these conclusions. For example, the POISE-3 clinical trial in 2022 studied the use of IV TXA (vs. placebo) and its effect on perioperative bleeding in noncardiac surgery [8]. Their primary efficacy outcome was a “composite bleeding outcome” referring to a combination of life-threatening bleeding, major bleeding and/or bleeding into a critical organ. The primary safety outcome was also a composite referring to a combination of MI, non-hemorrhagic stroke, peripheral arterial thrombosis, and symptomatic proximal venous thromboembolism. The POISE-3 trial also demonstrated reduced bleeding with TXA. While it did not demonstrate noninferiority with regards to its safety outcome, rates were not unequivocally inferior. Given its benefit in different types of surgery, the use of TXA has also been studied in different urologic procedures including radical cystectomy, percutaneous nephrolithotomy, and endoscopic surgery for BPH.

## TXA and Endoscopic Surgery for BPH

In this review, we will focus on the evidence pertaining to the use of TXA in endoscopic surgery for BPH. While endoscopic surgery for BPH is minimally invasive, perioperative bleeding in the form of significant hematuria remains a risk [9, 10]. Reducing significant hematuria has the potential benefit of reducing hospitalization, need for transfusion, and facilitating same day discharges when the procedures permit it. We were able to identify studies pertaining to the use of TXA in transurethral resection of the prostate (TURP), holmium laser enucleation of the prostate (HoLEP) and Aquablation. Other types of endoscopic BPH surgeries were not reliably evaluated.

### TURP

TURP has long been considered the gold standard of BPH surgery [11]. As such, several studies have examined the benefit of TXA in TURP. One such study was done by Rannikko et al. in 2004 [11]. In their RCT, 136 men who underwent TURP were randomized into a control group or a treatment group that received 2 g of TXA three times daily on the operative and first operative day. Patients taking finasteride or with a history of prostate cancer were excluded. Measured outcomes included surgical blood loss as measured by the amount of hemoglobin in the irrigating fluid, blood transfusion rate, and operative time. Their results demonstrated reduced bleeding when treated with TXA. This difference persisted when bleeding was indexed by amount of tissue resected. TXA also reduced operative time (36 vs. 48 min). Despite these benefits, they did not translate to lower transfusion rates or shorter hospital stay. In 2011, Kumsar et al. reported similar results in an RCT comparing TURP with TXA given intravenously (10 mg/kg) within the first half hour of the surgery [12]. A total of 40 patients were studied and evenly allocated in the treatment and control groups. IV TXA also demonstrated reduced perioperative bleeding when indexed by weight of prostate resected and shorter operative time. As with the previous study, Kumsar et al. were not able to demonstrate shorter hospitalization or duration of catheterization.

A more recent study by Vanderbruggen et al. specifically addressed the use of IV TXA with bipolar TURP [13]. In this RCT, 31 patients received IV TXA (10 mg/kg) at induction and a maintenance dose of (5 mg/kg/h) over 12 h compared to 34 patients who received saline as a placebo. In this study, perioperative bleeding as measured by hemoglobin loss, catheterization time, and hospitalization time were reduced in the treatment group. This study is notable for demonstrating reduced hospitalization and catheterization time which is inconsistent with the two previous described studies. One possible explanation is the use of continued maintenance infusion which was not used in the previous studies.

Another novel study by Rani et al. reported the use of TXA added to the irrigation fluid (1 g/5L glycine) during TURP [14]. Although TXA has not unequivocally been demonstrated to increase thromboembolism, its anti-fibrinolytic activity warrants caution in its use in patients who are already at higher risk of thromboembolic events. For this reason, TXA administered locally through irrigation solution provides a possibly safer route of administration. In this study, patients with a history of deep venous thrombosis and pulmonary embolism were excluded. A total of 60 men were evenly allocated to treatment and control group. Interestingly, topical TXA was sufficient to reduce blood loss indexed by prostate weight and improved visualization. No patients required transfusion, and all patients were automatically hospitalized until postoperative day 3 before trial of void and discharge, thus these outcomes could not be compared between treatment arms.

In 2021, Gupta et al. studied the use of combined IV TXA and TXA irrigation fluid in bipolar TURP [15]. Between January and November 2019, they recruited 70 patients to be evenly randomized to either 500 mg IV TXA and 500 mg TXA in 3 L of irrigation fluid vs. no TXA at all. As with previous studies, TXA administration reduced blood loss significantly as estimated by hemoglobin concentration in irrigation fluid. Overall, no differences were noted in operating time or hospitalization time until subgroup analysis was performed identifying benefit in prostate sizes of greater than 60 g.

Finally, in 2024 Diab et al. reported the use of intraprostatic TXA injections at the beginning of TURP to reduce perioperative bleeding [16]. In this randomized, double-blind trial, 60 patients undergoing TURP were recruited with weights between 50 and 80 g. Patients that were excluded were any patient at risk of thromboembolic event based on previous history or comorbidity, prostate cancer, use of 5-alpha reductase inhibitor, warfarin or aspirin. The 60 patients were evenly split into Group I which received 1 g of TXA dissolve din 50mL of 0.9% saline solution or Group II which received a 60 mL saline injection. Injection sites included the two lateral lobes at 5 o’clock and 7 o’clock, the median lobe and the apical tissue. Following the injections TURP was performed by a single surgeon using a 26Fr continuous flow resectoscope. The results of this study demonstrate similar outcomes to the previous studies mentioned using TXA via systemic administration routes. More specifically, Group I had shorter operative time (98.8 min vs. 108.3 min, *p* = 0.18), was rated to have a higher quality visualization, and lower early complication (< 24 h) rate of bleeding and clot retention. Irrigation fluid analyzed at the immediately and 6 h postoperatively demonstrated lower blood loss/hemoglobin levels in Group1, however this difference did not persist at 24 h postoperatively. As a result, hospital stay length did not differ either between groups.

### HoLEP

Laser enucleation of the prostate is typically performed with holmium laser (HoLEP) and has fast become a standard of care in endoscopic BPH surgery as a size independent procedure [17]. HoLEP has many benefits compared to TURP including better hemostatic control, closer anatomic resection, feasibility in larger prostates and as a same day procedure. In 2022, Assmus et al. studied the potential benefit of TXA with HoLEP. In this single blind RCT patients either received 1 g IV TXA or no treatment [18]. Patients who had any contraindication to TXA were excluded. A total of 110 eligible patients were identified and equally allocated between treatment and control arms. In this study, transfusion rate, duration of hospitalization, success of trial of void, and same day discharge was not impacted by using TXA.

Following the same line of questioning, Lee et al. performed a retrospective review of their HoLEP patients [19]. A total of 1037 HoLEP patients operated between August 2018 and November 2022 were included in their cohort. Patients who had a history of stroke, transient ischemic attack, or coronary stent placed within 18months of surgery were excluded. In this retrospective cohort, a total of 382 patients received 1 g of IV TXA at induction compared to 655 patients did not receive TXA. Baseline characteristics such as age, body mass index, history of prior BPH procedure, prior catheter placement, and prostate weight did not differ between cohorts. Diab et al. used rates of return to the operating room (RTOR) due to postoperative bleeding as their primary outcome. This outcome did not differ between patients who did and did not receive TXA, with rates being measured at 3.1% and 3.7%, respectively (*p* = 0.25). However, TXA administration was associated with lower transfusion rates (0.5% vs. 2.0% *p* = 0.016). TXA administration was not associated with lower catheter reinsertion or clotting complications.

The current evidence for use of HoLEP in TXA is currently lacking. While we have cited one RCT and one retrospective study, the conclusion that TXA may not benefit clinical outcomes in HoLEP is not surprising given its already low bleeding rates.

### Aquablation

Aquablation is one of the newest endoscopic surgeries to enter the armamentarium of BPH surgeries [20]. Using a robotic console, Aquablation leverages the use of high-pressure waterjet technology to remove adenomatous tissue while preserving the verumontanum and ejaculatory ducts. Similar to HoLEP, Aquablation has been shown to be effective in both smaller and larger glands, but with a much higher bleeding risk. In the initial trials of Aquablation bleeding risk was cited at around 10%. Currently, cautery loop hemostasis after Aquablation is used to reduce the bleeding risk to about 2% [20]. Given the higher associated bleeding risk, the application of perioperative TXA is of interest to reduce operative time or obviate the need of post-ablation cautery. While no formal study has been published, Eid et al. recently presented preliminary data of their clinical trial using TXA concurrently with Aquablation [21]. Between August 2022 and June 2024, 20 patients were randomized to surgery with TXA or without. Their results demonstrated significantly smaller reduction in hemoglobin levels when Aquablation is performed with TXA. This, however, did not change operating time significantly. No other outcomes were reported and final results are pending.

## Summary and Future Directions

Within the context of endoscopic surgeries for BPH, TXA appears to have the most benefit when performing TURP. More specifically, administration of TXA consistently reduces blood loss during TURP independent of administration route. However, this did not consistently correlate to improvement of clinical outcomes such as transfusion rate, operative time, catheterization time or hospitalization time reducing its clinical impact. Meanwhile, the use of TXA in other endoscopic BPH surgeries is largely limited with some evidence emerging in its use with HoLEP. While the latter studies in HoLEP have demonstrated little benefit, studies in patients at higher bleeding risk or larger prostates merits interest. Indeed, consideration should be made to the potential use of locally administrated TXA in patients who may not be able to suspend anticoagulation but still undergo HoLEP. The low absorption associated with TXA administered locally may allow for the local anti-fibrinolytic effect while avoiding the potential increased risk of thromboembolism associated with systemic absorption. Finally, while TXA has been the anti-fibrinolytic of choice in recent years due to its safety profile and low cost, other anti-fibrinolytics are available and worth studying.

## Conclusion

Within the context of endoscopic surgeries for BPH, TXA appears to have the most benefit when performing TURP. More evidence is required to conclude on the benefit in other types of BPH surgery including HoLEP.

## Key References

Diab, T., et al., *Intraprostatic Injection of Tranexamic Acid Decrease Blood Loss During Monopolar Transurethral Resection of the Prostate: A Randomized Controlled Clinical Trial.* Urology, 2024. **191**: p. 119–126.

- Side effects related to TXA appear to be related to its systemic administration; in this study Diab et al. present favorable results from TXA administered locally through an intraprostatic injection during TURP.

Assmus, M.A., et al., *Tranexamic Acid Does Not Improve Outcomes of Holmium Laser Enucleation of the Prostate: A Prospective Randomized Controlled Trial.* J Endourol, 2023. **37**(2): p. 171–178.

- TXA appears to have little benefit when used during HoLEP due to the procedures innate hemostatic control.

Eid, N. and J. Hillelsohn, *(219) the Impact of Tranexamic Acid on Hemoglobin Levels and Operating Time in Aquablation for Benign Prostatic Hyperplasia: Preliminary Results from a Retrospective Case–Control Study.* The Journal of Sexual Medicine, 2024. **21**(Supplement_7).

- Application of TXA into emerging technologies such as Aquablation require more study.

## Data Availability

No datasets were generated or analysed during the current study.
